# Constructing training set using distance between learnt graphical models of time series data on patient physiology, to predict disease scores

**DOI:** 10.1371/journal.pone.0292404

**Published:** 2023-10-19

**Authors:** Dalia Chakrabarty, Kangrui Wang, Gargi Roy, Akash Bhojgaria, Chuqiao Zhang, Jiri Pavlu, Joydeep Chakrabartty

**Affiliations:** 1 Department of Mathematics, Brunel University London, Uxbridge, United Kingdom; 2 Department of Computer Science, University of Warwick, Coventry, United Kingdom; 3 Department of Haematology, HealthCareGlobalEKO Cancer Hospital, Kolkata, India; 4 Hammersmith Hospital, Catherine Lewis Centre, London, United Kingdom; University Magna Graecia of Catanzaro, ITALY

## Abstract

Interventional endeavours in medicine include prediction of a score that parametrises a new subject’s susceptibility to a given disease, at the pre-onset stage. Here, for the first time, we provide reliable learning of such a score in the context of the potentially-terminal disease VOD, that often arises after bone marrow transplants. Indeed, the probability of surviving VOD, is correlated with early intervention. In our work, the VOD-score of each patient in a retrospective cohort, is defined as the distance between the (posterior) probability of a random graph variable—given the inter-variable partial correlation matrix of the time series data on variables that represent different aspects of patient physiology—and that given such time series data of an arbitrarily-selected reference patient. Such time series data is recorded from a pre-transplant to a post-transplant time, for each patient in this cohort, though the data available for distinct patients bear differential temporal coverage, owing to differential patient longevities. Each graph is a Soft Random Geometric Graph drawn in a probabilistic metric space, and the computed inter-graph distance is oblivious to the length of the time series data. The VOD-score learnt in this way, and the corresponding pre-transplant parameter vector of each patient in this retrospective cohort, then results in the training data, using which we learn the function that takes VOD-score as its input, and outputs the vector of pre-transplant parameters. We model this function with a vector-variate Gaussian Process, the covariance structure of which is kernel parametrised. Such modelling is easier than if the score variable were the output. Then for any prospective patient, whose pre-transplant variables are known, we learn the VOD-score (and the hyperparameters of the covariance kernel), using Markov Chain Monte Carlo based inference.

## 1 Introduction

It is highly desirable for medical practitioners to avail of a capacity for prediction at the pre-onset stage, of the risk of onset and progression of a disease in a new patient, using data on parameters that inform on this patient’s predisposition to the given disease. Such information potentially helps to undertake early intervention when the onset risk is predicted as high. The desired prediction of the risk parameter in a prospective patient is possible via the learning of the functional relationship between the vector of pre-onset variables, and a variable that parametrises or scores such risk of onset of the disease. It is only if this functional learning is rendered possible, that prediction of the risk score is rendered possible, at the known pre-disposition parameters of the new patient. Here we recall that the pre-requisite for supervised learning of a function that represents the relationship between a pair of variables, is training data on the input-output pairs. In our problem, one of this pair of variables is the vector of pre-disposition parameters, and the other is the score of the risk of disease onset. So, unless values of this score are accessible for each patient—whose predisposition parameters are known—the sought training set will remain out of reach. In other words, the automated prediction of the score that informs doctors about susceptibility of a prospective patient to a (considered) disease will remain elusive, unless we find a way to generate the value of such score for each patient in a retrospective cohort, thereby generating the desired training dataset.

In this paper, we discuss a method for predicting the risk score for a new patient of certain blood cancers, to develop the disease called Sinusoidal Obstruction Syndrome/Veno Occlusive Disease (or VOD hereon) that often sets in, following a Haematopoietic Stem-Cell Transplantation (HSCT), that a patient has undergone as an attempted cure of their underlying blood cancer [1–6, 13]. VOD causes constriction of the veins in a patient’s body [6, 7], causing possible malfunctioning of different vital organs, such as the lungs, kidneys, central nervous system, etc. [13]. Hepatic VOD is a common manifestation of this disease and we will refer to this manifestation, when we speak of VOD below, unless stated otherwise. We will also refer to HSCT below as a “bone marrow transplant” or simply as “transplant”.

VOD is a life-threatening complication that often follows bone marrow transplants, with mortality rates reported as ≤ 80%, [8–12]. Owing to the high mortality rates of this disease, VOD management demands frequent examination of symptoms, though there exist multiple conditions that “mimic VOD/SOS”, rendering “real-life differential diagnosis a true challenge” [13]. This is echoed by [14] and by [15] who advocates a “high index of suspicion” that they say “is needed to diagnose hepatic SOS”. Diagnosis of VOD is traditionally monitored using the Baltimore or the modified Seattle criteria, while some of the shortcomings of these criteria have been addressed via a new diagnostic criterion and a scale for severity grading of suspected VOD that have been advanced by the European Society for Blood and Marrow Transplantation [16, 17]. Crucially, [6, 16, 18–20] reiterate the proposal of [9], to emphasise that early diagnosis and treatment are positively correlated with survival, as do [13]. This motivates the need for prediction of the risk of a prospective patient to develop VOD, at the pre-transplant stage. We quantify this risk in the form of the VOD score that we will predict at the pre-transplant stage for a new transplant candidate, given their pre-transplant parameters that will inform on this patient’s underlying cancer; co-morbidities; relevant demographic and clinical parameters.

In lieu of an objective score for VOD onset and development, it may appear possible to undertake the learning of the pattern in the data collected from a retrospective set of bone-marrow transplant recipients, whose sufferance of VOD has been identified using a given model or interpretation of severity categorisation. We will need to review such attempts at using standard machine learning (ML) tools, [21], in the context of the question that Haematologist-Oncologists’ fundamentally desire an answer to, at the pre-transplant stage, namely: “how severely will VOD develop in a prospective patient?”. The efficacy of the reported ML tools in providing a reliable and explainable (and preferably continuous-valued) quantification of the virulence of VOD progression in the prospective patient will need to be reviewed, against any “black boxed” facility that lacks interpretability; lacks generalisability—to other implementation of VOD mitigation and HSCT protocol parameters—in spite of the large sample size, (as flagged up in the critical review by [22]). Such ML tools also typically perform inaccurate predictions; definitely fail in predictions when asked to extrapolate beyond the convex hull of the training set; and predict on the basis of potentially incorrect labelling of the target variable. So a review of this approach will be undertaken shortly in Section 2. There we will recall the nuances that challenge general automated predictive modelling, [23–25], in addition to criticisms of the usage of ML approaches within VOD prediction, [22, 26].

Indeed, in such an ML-based approach, there is no objective VOD-score that is made available, but these ML-based approaches use the categorised VOD status of patients in a retrospective cohort as the target variable, i.e. these approaches use the VOD status of patients who have already undergone the transplant. Such a VOD status is elicited by physicians, but this suffers from the problem that it is not sufficiently unambiguous, given the (aforementioned) possible conflation of symptoms of VOD and other underlying diseases [13, 15]. Irrespective of the diversity of the machine learning tools that are employed to learn the relationship between pre-transplant vector variable and the VOD score variables, (though the usable techniques reported here are ensemble methods), these fundamental shortcomings of the ML approaches leave us wanting for a reliable prediction of the VOD score of a prospective candidate for HSCT, at the pre-transplant stage.

Our work learns the continuous score of each retrospective patient, in an automated and entirely data-driven way, that is integrated for all levels of severity of the disease, to then learn the sought relationship between this score variable and the vector of pre-transplant variables. Ultimately, we predict the uncertainty-included real-valued score of a prospective patient at the pre-transplant stage. The main advantage of our method lies in the fact that we can predict the VOD-score before the transplant—thus enabling the crucially-helpful early intervention—by using only the data that are available. Patients identified to be more at risk of developing VOD after their HSCT, are then treated with VOD prophylaxis, namely, Defibrotide, [6, 19, 20, 27]. Defibrotide being expensive—at $500 per vial, of which two to three are required every day for adequate effect over the pre-transplant period of about eight days—is affordable for usage towards the mitigation of VOD onset only in those patients who are at high VOD risk. At the same time, such at-risk patients are monitored carefully post-transplant, to flag up any early signs of VOD onset. Importantly, we avoid reliance on the monitoring of symptoms for the grading of severity of the disease, since ambiguous manifestation of the relevant symptoms occurs commonly. We offer a reliable VOD-score that is uncertainty-included, while retaining capacity for acknowledging physicians’ priors towards a patient’s VOD status.

Once the (relative) VOD-scores are learnt for each patient in the retrospective cohort, we populate the training set comprising pairs of values of pre-transplant parameter vector and VOD-score, to learn the functional relation between these two variables, by modelling this function as a random realisation from a Gaussian Process (GP). In fact, the need for learning of far fewer hyperparameters of the kernel—that parametrises the correlation function of this GP—prompts our treatment of the scalar-valued VOD-score variable, (instead of the high-dimensional pre-transplant parameter vector), as the input to this sought function. This model choice leads to the need to learn the VOD-score at which the observed vector of pre-transplant parameters of the prospective-patient is realised; this is distinguished from the conventional closed-form prediction of the output of a GP-modelled function, at a test input [28].

[29] suggest the use of biomarkers for VOD identification. This however is not relevant to our work as we seek to predict the score that parametrises the risk for a patient at the pre-transplant stage, to develop VOD after they undergo the transplant. Indeed, identification of the gradation of VOD progression in a patient, using (multiple) biomarkers of their plasma, is relevant only at the post-transplant stage. While such biomarkers-based testing would be useful in lifting possible ambiguities about a patient’s VOD progression—thereby permitting confident diagnosis of VOD severity in each patient of a retrospective cohort—biomarkers-based testing demands infrastructure that is often out of bounds in multiple institutions. For example, some of the Haematologist-Oncologists in our team did not have access to the infrastructure that permits biomarkers-based testing of bone-marrow transplant recipients.

The paper is organised as follows. Section 2 discusses existing work in the context of predicting VOD progression in a prospective patient. Thereafter, we discuss our model in Section 3, which leads us to Section 4, in which we put forward the learning of the VOD-progression score of a prospective patient. Following such discussion, our results are presented in Section 5, while in Section 6, the ranking of the various risk factors for VOD is reported, in order of the potency of influence on VOD-progression. We conclude the paper with Section 7.

## 2 Our work in the context of existing work

[21] report on the results of the prediction of VOD status in a prospective recipient of HSCT, by (supervised) learning of the pattern in the data comprising observations on 20 selected features (as the inputs), and the severity category (as a target variable), using a variety of machine learning tools, for three different models or definitions that they invoke to assess VOD status in each of about 2500 HSCT recipients in a retrospective set. These models respectively ask: whether VOD did onset in a patient in this retrospective set or not; whether severe-to-very severe VOD happened to any such patient or not; and whether “early death” was noted in a patient or not. Then it is clear that this approach cannot provide an answer to the question of how severe VOD will be in a prospective patient; instead, it answers separate questions, such as: “will the prospective patient develop VOD?”; “will the prospective patient develop severe-to-very severe VOD?”; “will the prospective patient suffer from early death due to VOD onset and development?”.

These questions will not directly answer the question framed above to replicate the straightforward query that the Haematologist-Oncologist desires a response to. The deviation of the answers that are potentially available from these ML approaches, from the answer desired by physicians at the pre-transplant stage, stems exactly from what [22] identify as the drawback of these ML approaches, namely that these methods “oversimplify clinical questions by dichotomizing outcomes”; see also [23, 24]. While acknowledging this need to avoid such drawbacks, we state that interpretation of the severity level of VOD would be that much clearer, if the Oncologist could be provided a continuous, real-valued VOD onset + development score for any prospective patient, which for example, if lower than a cutoff, will indicate that VOD will not set in, in this patient, while a value higher than an identified threshold could be identified as severe VOD. Additionally, for clarity of comparison amongst patients, we realise that the VOD-score variable—if categorised—should ideally be on an equidistant scale. Else, usage of mean of such scores, as a central tendency, will be misplaced [30]. Basically, unless this scale is equidistant, a patient with a “high” score will in general need to be interpreted carefully in comparison to one who has a “low” score—and not as much at a higher risk of developing VOD, compared to another patient with a “moderate” score, as this patient is, compared to the patient with the “low” score.

Importantly, we provide a continuous real-valued VOD score in our method, where this score parametrises the risk for the given prospective patient to develop VOD within the monitored time interval. Our scores are offered within a scheme that is integrated across all levels of VOD susceptibility, and robust to variations in cohort size and features. We will demonstrate capacity of extrapolation in our predictions, i.e. even when the prospective patient’s pre-transplant features lie outside the convex hull of that of the retrospective patients in the training set, we are able to sucessfully predict the VOD score in our Bayesian approach, using even weak priors centred on the physicians’ opinions.

The diverse machine learning tools that [21] use to perform automated prediction in test cases, include logistic regression; Naïve Bayes; bagging techniques such as Random Forest; and boosting techniques such as Extreme gradient boosting (XGBoost) and Adaboost. Of these, XGBoost was noted to achieve optimal accuracy of prediction. Subsequent to cross-validation, the Receiver Operating Characteristic Area Under the Curve (ROCAUC) was computed to inform on the accuracy of the undertaken classification, for each considered model of categorisation. This was reported as 0.750 when VOD onset—or not—was the model of the categorisation; 0.778 when severe to very severe VOD onset was the categorisation model; and 0.738 for early death—or not—was used as the caegorisation. These ROCAUC values are not good for any of the three criteria, even with the best-performing learning algorithm, prompting the need to improve accuracy. Our presented method offers much higher accuracy.

The interpretability issue that [22] rightly hold machine learning “black boxes” to be deficient in, is available in our model, where every step is lucidly interprtable and understandable. Lack of generalisability of machine learning results is another problem, [22]. Following on from the critical review by [22], usage of “a large dataset does not necessarily mean” that the method “can be applied to different datasets (i.e., not generalizable to a different time and/or location)”. Multi-cohort and multi-institutional generalisability to cover for unevenness in VOD prophylaxes used in different countries is the major aim of our work, and we demonstrate this partially here, via our multi-cohort and multi-institutional application.

Again, the very construction of the training set used in machine learning approach, is in itself questionable, since the diagnosis of VOD and its intensity is not unambiguous as suggested in the literature [13, 15], and likely to not be similarly unambiguous across cohorts/institutions. So using such diagonised labels as the target variable, could induce errors.

## 3 Model

For a retrospective-patient, the pre-transplant attributes are recorded, in addition to their physiological parameters. The latter parameters include blood pressure, body temperature, capillary saturation, etc., and these parameters are recorded from a pre-fixed time point before the transplant, to a chosen time after the transplant, though not all patients survive this full time interval, given the potentially-terminal nature of the diseases that such patients are afflicted with. However, a prospective-patient is one who is being considered at the pre-transplant stage, so that their pre-transplant attributes are recorded, but no time series data on their physiological parameters is available at this pre-transplant stage.

We anticipate using the information available on physiological parameters of the considered retrospective-patients, to learn the VOD-score of each such patient. Once we are able to learn such a VOD score, we will populate the pair: value of a patient’s pre-transplant attribute vector, and their learnt VOD-score. Doing this for all retrospective-patients, will populate the originally-absent training set that is a requisite for the supervised learning of the relationship between a patient’s pre-transplant attribute, and their VOD score. So once this training set is generated, we will pursue the supervised learning of the relation between the pre-transplant attribute and VOD score—by modelling their relationship with a Gaussian Process. Having modelled this relationship as a function that is sampled from a Gaussian Process, we will then predict the VOD-score in a prospective-patient, given their known pre-transplant attributes. However, we appreciate that such eventual prediction (of the VOD score of a prospective patient) is only possible, if we can successfully initiate the learning+prediction strategy that we have delineated here. In other words, we can predict the VOD score of a new patient, only if we have been able to use the information on physiological parameters of each retrospective patient, to inform on their respective VOD scores.

However, there is no reliable and robust model that offers the functional relationship between the temporally-evolving physiological parameters of a retrospective-patient, and their VOD-score. Imposing an *ad hoc* parametric form to said functional relationship will in general be a mistake, and will result in wrong predictions of the VOD-score for any patient in the cohort of retrospective patients, leading to an unreliable training set that is to be used in the prediction of the VOD-score of a prospective-patient. Observations of physiological parameters alone, can only allow for a physician-elicited, categorised parametrisation of the intentsity of VOD progression in a patient. But such elicited scores are insufficiently reliable, given the reported conflation of “signs and symptoms” of VOD with “other post-transplant complications” [14]; the same is suggested by [13, 15]. Additionally, there is of course no scope of reliable assignment of a continuous VOD-score on the basis of such observations and elicited scores.

In light of this worry, we formulate a new method to learn the relative VOD-score in any retrospective patient, using diversely long time series datasets on physiological parameters, where such time series data is available for each retrospective-patient. The time series data of one patient does not have the same temporal coverage as that of another patient in general, owing to differential patient longevities; hence our qualification of these time series datasets as “diversely long”. This then presents another challenge to the learning strategy that uses such time series data. Our methodology should be then robust to the length of the time series data.

To address such idiosyncracy of the data, we learn the relative VOD-score by computing a distance between a pair of graphical models, learnt given a pair of time series data sets of the respective pair of retrospective-patients. In our work, any such graphical model is built out of realisations of a random graph variable, that is constructed in a probabilistic metric space, such that each edge of a graph is a probability. Now, the measure of affinity between two nodes in this graph, is complimentary to the distance between these nodes. So in this graph, the mutual affinity is also a probability. In fact, the inter-nodal distance function is a cumulative probability distribution, corrected by a constant, and the inter-nodal affinity is also a probability corrected by a constant. This inter-nodal affinity is found to be given by the marginal probability of the edge variable that joins the considered pair of nodes. If in a sample of realisations of this random graph, the sample mean of the marginal probability of an edge, exceeds a pre-selected cutoff probability, the edge exists in our final graphical model; else it does not.

Given that the graph variable is random, we can define its probability distribution; so, we can compute a statistical distance (eg. the Hellinger distance) between the posterior probability densities of any of two random graphs, given the respective time series data. Such an inter-graph distance then informs on the difference in the correlation structure of the physiological parameters of one patient, compared to that of another, where such a difference in the correlation structures stem from the differential progress of VOD in the two patients. Thus, the usage of such an inter-graph distance, as a marker of the relative progress of VOD in a patient—renders the length of the time series data of the physiological parameters, irrelevant. In our work, the inter-graph distance between the graphical model of a patient, and that of an arbitrarily-chosen reference patient, models the score of VOD progression in this patient, relative to that of the reference patient.

Once the (relative) score variable is learnt using the aforementioned inter-graph distance, we model the functional relationship between this (relative) VOD score and the vector of pre-transplant parameters, by modelling this function as a sample function of a Gaussian Process (GP). To minimise the learning of the parameters that specify the covariance function of this GP, we suggest choosing the lower-dimensional of these two variables, i.e. the scalar-valued VOD-score variable, as the input of this function. This model choice renders the output of this function a vector, i.e. this sought function is then rendered vector-valued. Then modelling such a function as the sample function of a GP implies that the invoked GP is vector-variate, such that the joint probability of a finite number of outputs of this function is then matrix Normal—a density that is parametrised by a vector-valued mean and two covariance matrices.

Of these two covariance matrices of the matrix Normal likelihood, the inter-column covariance matrix informs on the covariance between a pair of pre-transplant parameters. On the other hand, the inter-row covariance matrix is such, that an element of this matrix is the covariance between the pre-transplant variables of one patient, and those of another patient. Then any element of the inter-row covariance matrix can be parametrised using a kernel function, i.e. is modelled as a declining function of the difference between the pair of input values (i.e. VOD scores), the covariance between the outputs at which, is this considered element. The idea behind kernel parametrisation of a covariance function relies on the model that the further a pair of inputs, lower is the covariance between the outputs relevant at each such input. We choose a simple kernel, and learn the hyperparameters of this chosen kernel function, given the data.

As for the inter-column covariance matrix, we cannot undertake its kernel parametrisation since there is no identifiable “input” variable that the kernel could be defined as a function of. So we learn this matrix using the unbiased estimate of covariance between each pair of columns in the data comprising the pre-transplant parameters of the retrospective patients.

Thus, likelihood of these model parameters in our work is then rendered matrix Normal, as distinguished from the multivariate Normal density that would have resulted, had we chosen to learn a scalar-valued function. We argue that the comparatively more complicated logistics of this model—over the model that uses the vector of pre-transplant variables as the input—are outdone by the ease of making inference upon far fewer kernel hyperparameters in this model, (using Markov Chain Monte Carlo, or MCMC techniques). Importantly, difficulties with the mixed nature of the pre-transplant variables are also mitigated by considering these variables to comprise the output—than the input. Whenever the VOD-score of a prospective patient is sought, we will learn anew, the hyperparameters of the kernel that parametrises the inter-row covariance matrix, simultaneously with the VOD-score of this prospective patient.

Crucially, we also rank the pre-transplant attributes by potency of its effect on the VOD-score.

### 3.1 Model details

Let the *j*-th retrospective-patient’s physiological parameter vector **Θ**, observed a time point *T* = *t*_*i*_, be $\theta_{t_{i}}^{\left(j\right)}$, where $\Theta \in H\subset R^{m}$, i.e. there are *m* number of physiological parameters that are observed for any patient. Thus, **Θ** = (Θ_1_, …, Θ_*m*_)^*T*^. There are *N*_*p*_ number of retrospective-patients in the observed data set, such that (s.t.) *j* = 1, …, *N*_*p*_. Observations of physiological parameters are undertaken from the pre-transplant time point *t*_*min*_ to a post-transplant time point $t_{max}^{\left(j\right)}$, for the *j*-th patient; $t_{max}^{\left(j\right)}\leq t_{max}$, where observations are maximally taken till post-transplant time *t*_*max*_, though the *j*-th patient may survive to $t_{max}^{\left(j\right)}$. So $t_{i}\in \left[t_{min},t_{max}^{\left(j\right)}\right]$.

The time series data on the *m* physiological parameters, of the *j*-th retrospective patient, available during the time interval $\left[t_{min},t_{max}^{\left(j\right)}\right]$ is then represented as the data matrix $D_{j}≔\left(\theta_{t_{min}}^{\left(j\right)}\vdots \ldots \vdots \theta_{t_{max}^{\left(j\right)}}^{\left(j\right)}\right)$. Thus, for the *j*-th patient, this data matrix **D**_*j*_ is $(t_{max}^{\left(j\right)}-t_{min}+1)\times m$-dimensional; *j* = 1, …, *N*_*p*_.

Thus, data matrices of all retrospective patients share a common number of columns and a varying number of rows, driven by the patient’s longevity.

Pre-transplant parameters are observed for both retrospective-patients, as well as prospective-patients. Let the pre-transplant parameter vector ***Y*** be *d*-dimensional s.t. $Y\in Y\subset R^{d}$. Some components of the pre-transplant parameter vector ***Y*** are numerical in nature, while others are binary, and still other pre-transplant parameters are categorical taking values at more than two levels. We replace a categorical pre-transplant parameter that takes values at *k* categories, with *k* − 1 number of binary dummy covariates as per the standard practice. For example, the categorical variable of “Cancer type”—which can take values of “Acute Lymphoblastic Leukaemia (ALL)”; “Acute Myeloid Leukaemia (AML)”; “Aplastic Aneamia”; “Chronic Myeloid Leukaemia (CML)”; “Myelofibrosis”; “Other”—is replaced by 5 binary variables called “ALL”, “AML”, “Aplastic”, “CML”, and “Myelofibosis”, s.t. if the “Cancer type” in any patient is of the “Other” class, then each of these binary variables take the value of 0.

We use time series data **D**_*j*_ of the *j*-th retrospective patient, to learn the graphical model of this dataset, ∀*j* = 1, …, *N*_*p*_. Here, any such graphical model is learnt using realisations of a Random Geometric Graph (RGG) [31, 32] drawn in probabilistic metric space [33, 34], rendering it a Soft Random Geometric Graph (SRGG).

### 3.2 RGG in a probabilistic metric space

A Random Geometric Graph (RGG) is one in which the edge between any pair of nodes exists only if the mutual distance between these two nodes falls below a threshold distance *τ* > 0. In a Soft Random Geometric Graph (SRGG) such uncertainty on the edge variable between any nodal pair is acknowledged, as is the uncertainty on the location of any node; distance between two nodes is then a random variable (*D* ≥ 0) and probability that the edge between two nodes exists, is given by the “connection function” *ϕ*(*D*), where $\phi :R_{\geq 0}\to \left[0,1\right]$.

A probability metric space (H,F,Δ) is s.t. any point in it is a random variable in sample space H, and for any of 2 points $\Theta_{k},\Theta_{\ell }\in H$, we can define the cumulative distribution function (*cdf*) $F_{\Theta_{k},\Theta_{\ell }}\left(s\right)\in F_{+}$ of a “disparity” parameter *S*_*k*,*ℓ*_ ≥ 0 between these 2 points s.t. *S*_*k*,*ℓ*_ = 0 ⇔ *k* = *ℓ*. Thus, $F_{\Theta_{k},\Theta_{\ell }}\left(s\right)\equiv F_{S_{k,\ell }}\left(s\right)$. Here, $F_{+}$ is the space of *cdf*s with non-negative support. Also, Δ is defined as a binary operation—on $F_{+}$—that is commutative, associative, and has an identified identity.

We learn a graph as an RGG that is constructed in a native probabilistic metric space, s.t. the length of an edge between any two nodes of such a graph—i.e. the mutual “distance”—is a probability; these two nodes are associated with two random variables in H. Then, any inter-nodal distance, (offset by a constant), is a probability, while the probability of the edge to exist between these nodes, is identified with the complimentary affinity measure (offset by another constant) between these variables that are associated with these nodes. This edge probability is in fact the (posterior) probability of the edge variable, given the partial correlation *R*_*k*,*ℓ*_ between the random variables Θ_*k*_ and Θ_*ℓ*_ associated with the *k*-th and *ℓ*-th nodes respectively. We now discuss the partial correlation matrix and the edge marginal.

The partial correlation matrix **R** = [*r*_*k*,*ℓ*_] is computed given the inter-column correlation matrix **Σ** = [*σ*_*k*,*ℓ*_] as

$$R_{k,\ell }=-\psi_{k,\ell }/\sqrt{\psi_{k,k}\psi_{\ell ,\ell }},\;k\neq \ell ,\;\text{and}\;R_{k,k}=1\;\text{for}\;k=\ell ,$$

where the precision matrix is **Σ**^−1^ = [*ψ*_*k*,*ℓ*_].

### 3.3 Graphical model of physiological parameters

We motivate the *pdf* of |*R*_*k*,*ℓ*_|—the absolute of the *k*, *ℓ*-th partial correlation variable—given the *k*, *ℓ*-th edge parameter *G*_*k*,*ℓ*_ and the *k*, *ℓ*-th variance parameter *υ*_*k*,*ℓ*_, as $N(g_{k,\ell },\upsilon_{k,\ell })$ to account for the limiting conditions that at *G*_*k*,*ℓ*_ = *x*, density of |*R*_*k*,*ℓ*_| is highest for |*r*_*k*,*ℓ*_| → *x*, where *x* = 0, 1, Similarly, at *G*_*k*,*ℓ*_ = *x*, density of |*R*_*k*,*ℓ*_| is lowest for |*r*_*k*,*ℓ*_| → 1 − *x*. Here *υ*_*k*,*ℓ*_ is the scale that controls the decline in the density as |*r*_*k*,*ℓ*_| moves away from *g*_*k*,*ℓ*_. The above holds ∀*k* ≠ *ℓ*, *k*, *ℓ* ∈ {1, …, *m*}.

Then using uniform U[0,1] prior on *υ*_*k*,*ℓ*_, and Bernoulli(0.5) prior on *G*_*k*,*ℓ*_, we write the joint posterior probability density of *G*_*k*,*ℓ*_ and *υ*_*k*,*ℓ*_ given the observation on *R*_*k*,*ℓ*_. Thereafter, we marginalise *υ*_*k*,*ℓ*_ out of this joint posterior, to attain the marginal posterior of the *k*, *ℓ*-th edge variable as

$$m\left(G_{k,\ell }|r_{k,\ell }\right)=K^{/}[\sqrt{\frac{2}{\pi }}\;\text{exp}\;(\frac{-{\left(S_{k,\ell }\right)}^{2}}{2})-\left|S_{k,\ell }\right|\text{erfc}\;(\frac{|S_{k,\ell }|}{\sqrt{2}})],$$

where *S*_*k*,*ℓ*_ ≔ |*G*_*k*,*ℓ*_ − |*r*_*k*,*ℓ*_|| ∈ [0, 1].

The *pdf* of the observable *R*_*k*,*ℓ*_ motivated above is of course, not uniquely Normal; a smooth function that declines symmetrically about the mean of *G*_*k*,*ℓ*_, will be usable. Essentially, the only information that we possess on this density, are used in the design of this density, but the availability of such informaton is not sufficient to specify the form of the *pdf* further. However, we seek a form of the edge marginal that will help define the edge set of the sought graphical model; so the *pdf* of *R*_*k*,*ℓ*_ as a Normal suffices, and a form of the *pdf* of the observable *R*_*k*,*ℓ*_ that allows for a closed-form marginal posterior of the edge variable, is desired. Indeed, that closed-form edge marginal will be specific to the Normal form of the *pdf* of the observable, given model parameters—as used in our work. Bayesian inference on the edge variable *G*_*k*,*ℓ*_ from diverse choices of this *pdf*, is however likely to converge in mean, though the uncertainty learnt on the inferred value of this edge variable will vary as the chosen form of this *pdf* is made to vary. Then treating the parametrisation of the uncertainty as a random variable—such as the variance parameter *υ* of our chosen Normal form of the *pdf* of *R*_*k*,*ℓ*_—helps address the effect of varying the chosen form of this *pdf*. Eventually, it is the marginalisation over all values of this variance parameter that provides the edge marginal, ∀*k* < *ℓ*; *ℓ* = 2, …, *m*. Thus, the learning of the graphical model is not sensitively dependent on the choice of a particular form of the conditional *pdf* of *R*_*k*,*ℓ*_. Since the edges occur idependently of each other, the joint posterior of all edges, conditional on the (partial) correlation matrix, is the product of the edge marginal *m*(*G*_*k*,*ℓ*_|*r*_*k*,*ℓ*_) given above, over all nodal pairs.

The aforementioned marginal posterior of the variable *G*_*k*,*ℓ*_ that is the edge between the *k*-th and *ℓ*-th nodal pair—conditional on the partial correlation *R*_*k*,*ℓ*_ = *r*_*k*,*ℓ*_ between variables Θ_*k*_ and Θ_*ℓ*_—is sampled from, using Rejection Sampling. We generate *N*_*iter*_ such samples, and let the sample generated in the *q*-th iteration be ${\left\{g_{k,\ell }^{\left(q\right)}\right\}}_{k<\ell ;\ell =2}^{m}$. Then the SRGG variable defined on vertex set ***V*** is updated, via the updating of the edge set $E^{\left(q\right)}={\left\{g_{k,\ell }^{\left(q\right)}\right\}}_{k<\ell ;\ell =2}^{m}$. Thus, the edge set of the SRGG variable is updated ∀*q*.

Then using the edge sets $E^{\left(1\right)},E^{\left(2\right)},\ldots ,E^{\left(N_{iter}\right)}$, we construct the sample estimate of the marginal probability of the edge variable *G*_*k*,*ℓ*_, as

$$\hat{m}\left(g_{k,\ell }|r_{k,\ell }\right)=\sum_{q=1}^{N_{iter}}\left(g_{k,\ell }^{\left(q\right)}\right)/N_{iter},$$

which is the relative frequency for $G_{k,\ell }^{\left(q\right)}$ to be 1. This sample estimate $\hat{m}\left(G_{k,\ell }|r_{k,\ell }\right)$ of the edge marginal probability is computed for all *k*, *ℓ* pairs. As long as this estimated marginal probability of the *k*, *ℓ*-th edge exceeds the cutoff (or the threshold) probability *τ*, the edge exists in the final “graphical model” $G_{m_{j},\tau }^{\left(j\right)}\left(R_{j},V\right)$ of the *j*-th patient; else it does not.

Graphical model $G_{m_{j},\tau }^{\left(j\right)}\left(R_{j},V\right)$ is defined on the vertex set ***V***, for the partial correlation matrix $R_{j}=\left[r_{k,\ell }^{\left(j\right)}\right]$ and edge marginal $m_{j}\equiv \hat{m}\left(\cdot |r_{\cdot ,\cdot }^{\left(j\right)}\right)$, on edge set

$$E={\left\{g_{k,\ell }:g_{k,\ell }=1\;\text{if}\;\hat{m}\left(g_{k,\ell }|r_{k,\ell }^{\left(j\right)}\right)\geq \tau ;\;\text{else}\;g_{k,\ell }=0\right\}}_{k<\ell ;\ell =2}^{m}.$$

In the *q*-th iteration of Rejection Sampling, the random graph defined on vertex set ***V***, is $G^{\left(q\right)}\left(R_{j},V\right)$. The edge set ***E***^(*q*)^ = {*g*_*k*,*ℓ*_}_*k*<*ℓ*;*ℓ*∈{2,…,*m*}_ of $G^{\left(q\right)}\left(R_{j},V\right)$ is s.t. *g*_*k*,*ℓ*_ will be 1 or 0. Here *q* = 1, …, *N*_*iter*_.

### 3.4 Inter-graph distance & VOD-score

At every trial or iteration of Rejection Sampling, undertaken using data sets **D**_*i*_ and **D**_*j*_, we record the posterior probabilities of the random graphs $G^{\left(q\right)}\left(R_{i},V\right)$ and $G^{\left(q\right)}\left(R_{j},V\right)$ that are iterated over, given the partial correlation matrices of the respective data sets. Let the posterior of the *j*-th patient’s random graph variable, computed at its value sampled in the *q*-th iteration—given the partial correlation matrix **R**_*j*_—be $\pi (G^{\left(q\right)}\left(R_{j},V\right)|D_{j})$.

We define a distance using the graphs sampled in the iterations of the two separate Rejection Sampling undertakings using two different patients’ data. In our work, this is the discretised Hellinger distance *δ*_*i*,*j*_ between the posterior probabilities of the two graph variables, given the respective data. To be precie, *δ*_*i*,*j*_ is the distance between the graphical models learnt given the time series data sets **D**_*i*_ and **D**_*j*_ respectively, where these datasets comprise information on the physiological parameters of the *i*-th and *j*-th patients. The squared discretised Hellinger distance is

$${\left(\delta_{i,j}\right)}^{2}=\sum_{q=1}^{N_{iter}}{\left(\sqrt{u_{i}^{\left(q\right)}}-\sqrt{u_{j}^{\left(q\right)}}\right)}^{2}/N_{iter},$$

where

$$u_{i}^{\left(q\right)}\equiv \text{ln}\left(\pi \left(G^{\left(q\right)}\left(R_{i},V\right)|D_{i}\right)\right)-B,$$

where the logarithm of the posterior that we obtain from the MCMC chain, is scaled by the scale

$$B≔\text{max}\{\phi :\phi =\text{ln}\left(\pi \left(G^{\left(q\right)}\left(R_{i},V\right)|D_{i}\right)\right),q\in \left\{1,\ldots ,N_{iter}\right\},i\in \left\{1,\ldots ,N_{p}\right\}\}.$$

Here *N*_*p*_ is the number of patients in the retrospective cohort and *i*, *j* ∈ {1, …, *N*_*p*_}.

*δ*_*i*,*j*_ is a distance function since it is non-negative; symmetric; obeys the triangle rule; and is 0 ⇔ *i* = *j*.

### 3.5 Learning relative VOD-scores

We arbitrarily choose one of the *N*_*p*_ retrospective patients as the “reference patient”, whom we assign a VOD-score of 1. Then the VOD-scores of all other patients are computed relative to this reference patient. Without loss of generality, let the 1st patient in the retrospective cohort be referred to as the reference patient. Then we compute the distance *δ*_1,*j*_ between the graphical models of the 1st, i.e. the reference patient, and the *j*-th retrospective patient, ∀*j* = 2, …, *N*_*p*_. We model the absolute difference between the VOD-scores of two retrospective patients to be proportional to the computed distance between the graphical models learnt given the respective time series data on the physiological parameters of these two patients. Then, if the VOD-score of the *j*-th patient is *S*_*j*_, upon setting the constant of proportionality to be unity, |*s*_*j*_ − 1| = *δ*_1,*j*_ which implies that either *δ*_1,*j*_ = *s*_*j*_ − 1 or *δ*_1,*j*_ = 1 − *s*_*j*_.

At the same time, the distance between the *i*-th and *j*-th retrospective patients’ learnt graphical models is *δ*_*i*,*j*_ = |*δ*_1,*j*_ − *δ*_1,*i*_|. Here *i* ≠ *j*, ∀*i*, *j* = 2, …, *N*_*p*_.

- Then for *s*_*j*_ < 1 and *s*_*i*_ < 1,
  - under Case 1, when (observed or) computed *δ*_1,*i*_ > *δ*_1,*j*_, *s*_*j*_ = *δ*_*i*,*j*_ + *s*_*i*_,
  - while under Case 2 of observed *δ*_1,*i*_ < *δ*_1,*j*_, *s*_*j*_ = *s*_*i*_ − *δ*_*i*,*j*_.
- Again, for *s*_*j*_ ≥ 1 and *s*_*i*_ ≥ 1,
  - in Case 1 when *δ*_1,*i*_ > *δ*_1,*j*_, *s*_*j*_ = *s*_*i*_ − *δ*_*i*,*j*_,
  - while in Case 2 of observed *δ*_1,*i*_ < *δ*_1,*j*_, *s*_*j*_ = *s*_*i*_ + *δ*_*i*,*j*_.
- Similarly, we address the cases that emanate from the possibilties of: *s*_*j*_ ≥ 1 and *s*_*i*_ < 1; *s*_*j*_ < 1 and *s*_*i*_ ≥ 1.

Thus, ambiguities exist in the values that *s*_*j*_ ∀*j* = 2, …, *N*_*p*_ can attain, given the data $D_{\delta }≔{\left\{\delta_{i,j}\right\}}_{i\neq j;i,j=1}^{N_{p}}$. Such ambiguities deter us from opting to compute *s*_*j*_ deterministically. Additionally, complexity of deterministic computation of *s*_*j*_ for *j* = 2, …, *N*_*p*_ rises superlinearly with *N*_*p*_, since internal consistency is required amongst all scores. In light of this, instead on deterministic computation of the scores, we learn $s_{2},\ldots ,s_{N_{p}}$ within an MCMC approach, given data **D**_*δ*_.

In the *t*-th iteration of the MCMC chain, we propose the value $s_{j}^{\left(⋆,t\right)}$ of *S*_*j*_ from a Normal proposal density, with a mean that is the current value $s_{j}^{\left(t-1\right)}$ as in the previous iteration, i.e. the *t* − 1-th iteration; variance of the proposal density is fixed via experimentation. Indeed in our strategy, the VOD-score of a patient can be positive or negative, and this prompts the choice of the Normal as the proposal density.

To formulate the likelihood of *S*_*j*_, we define the *pdf* of the observable *δ*_1,*j*_, given *S*_*j*_ = *s*_*j*_, as a Normal with a chosen variance, and a mean of *s*_*j*_ − 1 if *s*_*j*_ > 1, or 1 − *s*_*j*_ if *s*_*j*_ ≤ 1. Since this conditional *pdf*—and thereby the likelihood of *S*_*j*_—is computed in the *t*-th iteration, at the proposed value of *S*_*j*_, we know if the value at which this *pdf* is computed, exceeds 1 or not. Thus, in the *t*-th iteration, mean of this conditional *pdf* is identified (∀*j* = 2, …, *N*_*p*_), and thereby, the likelihood is computed as the product of such *pdf*s, over all *j* = 2, …, *N*_*p*_.

The choice of the Normal form of this *pdf* can be questioned; it is motivated by the limiting conditions that the density is maximised if *δ*_1,*j*_ equals the mean of this density, and that the density tends to 0, as the value of *δ*_1,*j*_ increasingly deviates from this mean. Of course any other bell-shaped form of this conditional density of the observable would have worked as well, though in the Bayesian inference paradigm that is relevant in our work on the score variables, the mean value of *S*_*j*_ that is inferred upon, will be robust to the form chosen for the *pdf* of the observable. This notwithstanding, the uncertainty learnt on *S*_*j*_ would vary as we vary the form of the *pdf* of the observable given this score variable. However, the information on *S*_*j*_ is high in this inferential exercise, and such uncertainties are expected to be low. We further enhance such information on *S*_*j*_ by designing priors that include information on how in the *t*-th iteration, the proposed value of *S*_*j*_ compares, to the proposed value of *S*_*i*_, (*S*_*i*_ ≠ *S*_*j*_). Thus, in the *t*-th iteration, parameters of the prior on *S*_*j*_, computed at its proposed value $s_{j}^{\left(⋆,t\right)}$ can be identified. For example, if proposed $s_{j}^{\left(⋆,t\right)}>1$ and proposed $s_{i}^{\left(⋆,t\right)}>1$, with observed *δ*_1,*j*_ > *δ*_1,*i*_, the prior is chosen as $N(s_{i}^{\left(⋆,t\right)}+\delta_{i,j},v)$, where the prior variance *v* is fixed by choice, and the prior mean stems from the result that under these conditions, *s*_*j*_ = *δ*_*i*,*j*_ + *s*_*i*_, (as stated above). In this way, the prior on *S*_*j*_ is selected, for each case that is relevant for the proposed values of *S*_*i*_ and *S*_*j*_, and the observed *δ*_*i*,*j*_, ∀*i* ≠ *j*, *i*, *j* ∈ {2, …, *N*_*p*_}.

## 4 Learning relationship between (learnt) VOD-score & pre-transplant variables

Learning VOD-scores $s_{1},\ldots ,s_{N_{p}}$ of the *N*_*p*_ retrospective patients, capacitates the desired supervised learning of the function that represents relation between the VOD-score variable *S* and the *d*-dimensional pre-transplant parameter vector ***Y*** that is observed for all retrospective patients, as well as any test (or prospective) patient whose VOD-score we would wish to predict. Then using the training data $D≔{\left\{\left(s_{j},y_{j}\right)\right\}}_{j=1}^{N_{p}}$ that materialises upon our learning of the originally-absent VOD-scores of the retrospective patients, we undertake the supervised learning of the functional link between ***Y*** and VOD-score, using Gaussian Ppocess (GP) based modelling. Then any undertaken kernel parametrisation of the covariance structure of the GP that generates this function, will require expressing the correlation between a pair of the output variables that are outputs at each of two designed input values. So we realise that if we employ ***Y*** as the input variable, (and *S* as the output) of the sought function, the covariance kernel will then need to be computed at each of two design input vectors, i.e. at each pair of values of ***Y***; also, hyperparameters of such a kernel will need to be learnt. In this approach, we will need to (minimally) learn *d* hyperparameters, in the (simplest) kernel, and the joint probability of the *N*_*p*_ outputs at the design inputs that live in the training set **D** will be multivariate Normal. Predictions of mean and variance of the output VOD-score of a test patient are then closed-form. However, we can lessen our learning-load, if instead of this approach, we set the output to be ***Y***, rendering the input variable *S*. Then in the simplest kernel, we will need to learn only one hyperparameter. In addition to the comparatively easier learning in this approach, (over the previous approach), we benefit from avoiding computing the kernel in terms of the components *Y*_1_, …, *Y*_*d*_ (pre-transplant parameters), which are mixed (binary and numerical) by nature.

However, in this approach, the joint of the *N*_*p*_ vector-valued outputs is matrix Normal, which renders coding heavier than in the other model. The main worry in this model pertains to the prediction of the input score *s*^(*test*)^ for a test patient with observed pre-transplant vector ***y***^(*test*)^. Indeed in this case, we will not predict value *s*^(*test*)^ of the input, but learn it—along with the kernel hyperparameters—using MCMC. To summarise, we will undertake the model in which ***Y*** is the output and *S* the input to the sought function $f:R\to Y\subset R^{d}$.

In the model of the relationship between *S* and ***Y*** as ***Y*** = ***f***(*S*), where the vector-valued function ***f***(⋅) is sought, we set f(·)∼GP(μ(·),K(·,·)), where ***μ***(·) and ***K***(⋅, ⋅) are the mean and covariance functions respectively of this vector-variate GP [35, 36]. Then by definition of this GP, the joint probability $\left[Y_{1},\ldots ,Y_{N_{p}}\right]$ of outputs at each of the *N*_*p*_ design inputs that populate the training set **D**, is matrix Normal, i.e. $\left[Y_{1},\ldots ,Y_{N_{p}}\right]=MN\left(\mu ,\Sigma_{Patient},\Sigma_{Y}\right).$ Here we set the mean matrix ***μ*** to a constant matrix, the *i*-th row of which is the mean of *Y*_*i*_ across the sample of *N*_*p*_ patients, (*i* = 1, …, *d*). The inter-patient covariance matrix is $\Sigma_{Patient}^{\left(N_{p}\times N_{p}\right)}$, while the inter-pre-transplant-variable covariance matrix $\Sigma_{Y}^{\left(d\times d\right)}$. Here, the *ij*-th element of the inter-patient covariance matrix **Σ**_*Patient*_ informs on the correlation between the *i*-th and the *j*-th patients’ pre-transplant parameter vectors; *i*, *j* = 1, …, *d*. The *bc*-th element of the inter-pre-transplant-variable covariance matrix **Σ**_***Y***_ informs on the correlation between the *b*-th and *c*-th components of ***Y***; *c*, *b* = 1, …, *d*.

There are *d* = 30 pre-tansplant variables that are observed; this implies that learning of distinct elements of **Σ**_***Y***_ would entail the learning of (30^2^ − 30)/2 parameters. Making inference on these parameters directly using MCMC, is an infeasible task. At the same time, there is no variable that can be considered as an input variable at which the *N*_*p*_-dimensional vector of values of *Y*_*c*_ for the *N*_*p*_ patients, is realised, for any *c* = 1, …, *d*. Distinguished from this, a *d*-dimensional vector of values of *Y*_1_, …, *Y*_*d*_ is realised at a given value of the VOD-score variable *S* of a patient. Thus, the *d* × *d*-dimensional inter-pre-transplant-variable covariance matrix **Σ**_***Y***_—an element of which informs on the correlation between a pair of pre-transplant variables—cannot be kernel parametrised, but the *N*_*p*_ × *N*_*p*_-dimensional inter-patient covariance matrix **Σ**_*Patient*_ can be kernel parametrised. So we will use the unbiased estimate of the covariance between any pair of pre-transplant variables, as an element of **Σ**_***Y***_. On the other hand, **Σ**_*Patient*_ = [*σ*_*ij*_] = [*K*(*s*_*i*_, *s*_*j*_)], where we choose the simple Square Exponential (SQE) kernel for *K*(⋅, ⋅), i.e. *K*(*s*_*i*_, *s*_*j*_) = *a* exp(−(*s*_*i*_ − *s*_*j*_)^2^/2*ℓ*^2^). We learn the 2 hyperparameters *a* and *ℓ*, given data **D**.

As suggested above, that ***f***(⋅) is modelled as a sample function of a vector-variate GP, implies that the joint probability density of the *N*_*p*_ outputs (realised respectively at each design input), is matrix Normal. But this density is the density of the data variable $D_{Y}≔\left(Y_{1},\vdots ,\ldots \vdots ,Y_{N_{p}}\right)$ on the output variable, conditional on the parameters of the model, at the given design points. So, this density is the likelihood of the kernel hyperparameters:

$$\begin{array}{c} \sqrt{{\left(2\pi \right)}^{-dN_{p}}|\Sigma_{Y}{|}^{-N_{p}}{\left|\Sigma_{Patient}\right|}^{-d}}\times \\ \text{exp}\;(-Tr(\Sigma_{Y}^{-1}{\left(D_{Y}-\mu \right)}^{T}\Sigma_{Patient}^{-1}\left(D_{Y}-\mu \right))/2), \end{array}$$

where data **D**_***Y***_ comprises values of ***Y*** at the chosen design points $s_{1},\ldots ,s_{N_{p}}$. We invoke adequate priors on the kernel hyperparameters, and multiply the same with this likelihood, to allow for the joint posterior probability density of these parameters given the data **D**_***Y***_ and the given design points. We then generate posterior samples using MCMC, allowing for the computation of the marginal posterior probability of each parameter given the data. The marginals then allow for the learning of the 95% Highest Probability Density credible region (HPDs) on each parameter, given the data.

Our interests are however ulterior to the learning of the kernel hyperparameters; indeed, we want to learn the value *s*^(*test*)^ of the VOD-score of a new (or prospective) patient, who is examined at the pre-transplant stage, s.t. their pre-transplant parameter vector is recorded as ***y***^(*test*)^. Thus, the augmented data on the outputs (**D**_*aug*_) now includes $y_{1},\ldots ,y_{N_{p}}$—from the retrospective (training) patients—as well as ***y***^(*test*)^. Thus, $D_{aug}=(y_{1},\ldots ,y_{N_{p}},$ ***y***^(*test*)^). Conditioning the density of variables $D_{aug},S_{1},\ldots ,S_{N_{p}}$ on the model parameters *ℓ*, *a* and on *S*^(*test*)^, reduces to the ratio of: the joint density of ***D***_*aug*_ and $S_{1},\ldots ,S_{N_{p}},S^{\left(test\right)}$, conditional on parameters *ℓ*, *a*, and the density of *S*^(*test*)^ conditional on *ℓ*, *a*, i.e.

$$f\left(D_{aug},s_{1},\ldots ,s_{N_{p}}|\ell ,a,s^{\left(test\right)}\right)=f\left(D_{aug},s_{1},\ldots ,s_{N_{p}},s^{\left(test\right)}|\ell ,a\right)/f\left(s^{\left(test\right)}|\ell ,a\right).$$

Setting the conditional density *f*(*s*^(*test*)^|*ℓ*, *a*) as Uniform over a chosen interval in the VOD-score, we write the logarithm of the density of the data variable given the unknowns as

$$\begin{array}{c} log(f_{D_{aug},S_{1},\ldots ,S_{N_{p}}|\ell ,a,S^{\left(test\right)}}\left(D_{aug},s_{1},\ldots ,s_{N_{p}}|\ell ,a,s^{\left(test\right)}\right))=\\ -Tr(\Psi_{Y}^{-1}{\left(D_{aug}-\mu_{aug}\right)}^{T}\Psi_{Patient}^{-1}\left(D_{aug}-\mu_{aug}\right))/2+\;\text{constant}, \end{array}$$

where **Ψ**_*Patient*_ is the (*N*_*p*_ + 1) × (*N*_*p*_ + 1)-dimensional inter-column correlation matrix of the *d* × (*N*_*p*_ + 1)-dimensional augmented data **D**_*aug*_. The inter-row correlation matrix of this augmented data **D**_*aug*_ is **Ψ**_***Y***_ which is *d* × *d*-dimensional, but elements of which are different from elements of **Σ**_***Y***_ owing to the added column in the augmented data, over the original data **D**_***y***_ on the output variable. The mean matrix of the augmented data matrix is also changed from that of **D**_***y***_, and is *d* × (*N*_*p*_ + 1)-dimensional. Our MCMC-based inference permits ignorance of the unknown constant that is added to the log likelihood.

Along with this likelihood, we invoke priors on the hyperparameters *ℓ* and *a*, and incorporate priors elicited by the Haematologist-Oncologists on *S*^(*test*)^, to formulate the joint posterior probability density $\pi (\ell ,a,s^{\left(test\right)}|D_{aug},s_{1},\ldots ,s_{N_{p}})$. We learn the marginals of each learnt parameter using MCMC, allowing for the learning of 95% HPDs on each learnt parameter.

## 5 Results

**Fig 1:**
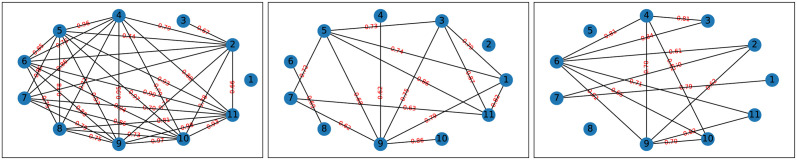
Graphical models of 3 patients in the retrospective cohort, (with the learnt mean relative score of 0.4374, 1 and 1.5526, from left to right) learnt given the time series data on the physiological parameters of each patient from a pre-transplant to a post-transplant time point. Parameter name is indicated at each node and the empirical probability for an edge to exist is marked on the edge. Only edges with probability > *τ* = 0.6 are included.

The multi-dimensional time series data on (*m* =)11 physiological parameters, and the data for (*d* =)30 pre-transplant parameters, for *N*_*p*_ = 25 patients in the retrospective cohort, was obtained by the Haematologist-Oncologists in our team; there was no inclusion-exclusion relevant to the recuitment of patients in the retrospective cohort, and data of all patients treated till the end of 2021 was included in this retrospective cohort. In Fig 1 we represent the learnt graphical models of three patients in this retrospective cohort. We used a cutoff probability *τ* = 0.6 to learn all graphical models; a different *τ* would affect the sparsity of the learnt graphical model, but would not have affected computation of scores of VOD progression, since it is the distance between the probability of the SRGG variable learnt using two datasets, that informs on scores. The graphical models in Fig 1 include that of the reference patient with an assigned VOD-score of 1, and two other patients, whose VOD-scores are learnt as higher and lower than 1 respectively. Hereon, all learnt VOD-scores are learnt relative to the score of 1 that is assigned to the arbitrarily-chosen reference patient, but we will not necessarily implement the adjective “relative” when we discuss a learnt score below.

### 5.1 Scores from multi-institution and multi-cohort patient data

If data on multiple retrospective cohorts are available, we first identify an arbitrarily-chosen patient as the reference patient of each such cohort. Thereafter, we compute the distance between the graphical model that is learnt for any patient in a cohort, and that for the pre-selected reference patient of this cohort. A reference patient in any cohort, is assigned a VOD progression score of 1, and on this scale, the score of any other patient in this cohort is computed, given the distance between their graphical model and that learnt for the reference patient of this cohort.

Next, we select the cohort—referred to as cohort*A*—the reference patient of which will be considered to be the reference patient over all the available cohorts. In other words, we choose the arbitrarily-selected reference patient of cohort*A* to be the “universal” reference patient.

Subsequently, upon learning the graphical model for each patient in each cohort, we compute the distance *d*_*A*,*B*_ between graphs learnt for the reference patient in cohort*A*, and that learnt for the reference patient of any other cohort—say, cohort*B*. Then relative to the reference patient of cohort*A*—who is arbitrarily assigned the VOD score of 1—the score of the reference patient of cohort*B* is 1 + *d*_*A*,*B*_ or 1 − *d*_*A*,*B*_, depending respectively on whether the reference patient in cohort*B* is identified by doctors to have VOD more progressed than the reference patient in cohort*A*, or vice-versa. In fact, it is only patients whose VOD progression status is clearly concludable by physicians, who are considered as possible candidates for the reference patient in cohort*B*.

So, relative to the score of 1 that is assigned to the “universal” reference patient—who we selected as the reference of cohort*A*—the VOD-score of the chosen reference patient in cohort*B* is changed from 1, to 1 + *d*_*A*,*B*_ or 1 − *d*_*A*,*B*_, as the case maybe, i.e. is shifted by *d*_*A*,*B*_ or −*d*_*A*,*B*_ respectively. This inter-reference-patient distance *d*_*A*,*B*_ is then used to adjust the distances computed for all patients in cohort*B*. To be precise, the relative VOD-score of each patient in cohort*B*, is now shifted by *d*_*A*,*B*_ or −*d*_*A*,*B*_, as the case maybe. This results in the score of any patient in cohort*B*, relative to the reference patient of cohort*A*, who is the reference acoss all cohorts, by our choice.

So data could be collected in different institutions, and/or at different times, but it is possible in our work, to use all such datasets, and assess VOD progression of all patients for whom data is available. In our work, we use time series data on physiological parameters of patients in three cohorts, who were monitored in different institutions.

**Fig 2:**
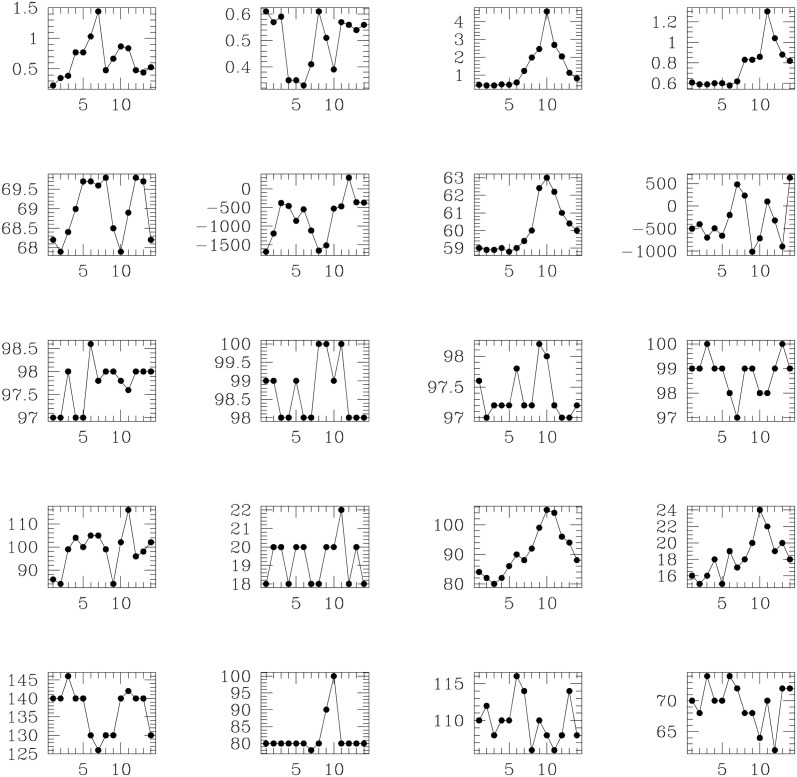
Plots of temporal variation of physiological parameters of two different patients—The 1st patient from Cohort 1 and the 1st patient in Cohort II. The temporal coverage of these plots extend from 8 days before the the bone marrow transplant, to 18 days after. The left-most two columns are those of the 1st patient in Cohort I while the two right-most columns depict parameters of the 1st patient in Cohort II. These physiological parameters are: systolic blood pressure—plotted in panels at positions (1,1) and (3,1) respectively for the patients in Cohort I and Cohort II; dystolic pressure plotted in panels at the (2,1) and (4,1) coordinates, for these two patients respectively; pulse rate for these patients in panels at (1,2) and (3,2) positions respectively; respiratory rate in panels at positions (2,2) and (4,2); body temperature in panels at positional coordinates (1,3) and (3,3); capillary saturation in panels at positions (2,3) and (4,3); body weight in panels at (1,4) and (3,4); fluid balance in panels at (2,4) and (4,4); total bilurbin in panels at positions (1,5) and (3,5); creatinine in panels at positional coordinates (2,5) and (4,5) respectively, for the patients in Cohorts I and II.

Such physiological parameters include body weight, body temperature, systolic and diastolic pressures, etc., amongst 11 parameters. These physiological parameters are recorded on multiple instances, for individual patients over a given time interval—namely about 8 days before the transplant to (maximally) 18 days after the transplant. However, not all patients survive this stipulated period of observation as they might succumb to an underlying disease before this full temporal interval is up. Thus, the data matrices comprising the time series measurements of the physiological parameters of different patients, will not have the same number of rows necessarily, though the number of columns of these matices is the same. Thus, the observed physiological information for the *j*-th patient in the *w*-th cohort is contained in an *n*_*j*_ × 11-dimensional matrix $P_{j}^{\left(w\right)}$; here *w* = *I*, *II*, *III*, *j* = 1, …, 5 for Cohort I; *j* = 1, …, 8 for Cohort II; *j* = 1, …, 12 for Cohort III. In Fig 2, variation of the physiological parameters with the time point of observation of 10 physiological parameters are depicted, for the 1st patient in Cohort I and the 1st patient in Cohort II. (The 1st patient in Cohort II was found in our work to have a higher risk score of SOS/VOD, than the 1st patient in Cohort I, who in fact did not develop VOD, as diagnosed post-transplant).

We learnt the VOD-scores of each of the 5 patients in Cohort I, relative to that of the 5-th patient of this cohort, who we arbitrarily assign the reference patient of this cohort. In Cohort II, we arbitrarily chose the 1st patient as the reference patient. The scores of all patients in Cohort III were learnt using the 12-th patient as the reference patient of this cohort. However, we desired that scores of all patients across the three cohorts be learnt relative to the score of one identified, “universal” reference patient. We chose the 1st patient of Cohort II as the “universal” reference patient of the whole “retrospective cohort”, by which we imply the cohort comprising all 25 patients from Cohort I, Cohort II and Cohort III, for each of whom, we have post-transplant information.

To achieve the scores of patients in Cohort I and in Cohort III, with respect to the “universal” reference patient—to be precise, the 1st patient in Cohort II—we proceed as suggested above. We compute the distance between the graphical models learnt for the erstwhile reference patient (number 5) of Cohort I, and the “universal” reference patient, as well as between the originally-selected reference patient (number 12) in Cohort III and the “universal” reference patient.

We computed the inter-graph distance *δ*_⋅,⋅_ between the reference patients of Cohort I (and of Cohort III), and the “universal” reference patient—who is 1st and the originally chosen reference patient of Cohort II—using information obtained from the doctors about

- whether VOD was more progressed in the originally-chosen reference (or 5th) patient of Cohort I, compared to the 1st patient in Cohort II (i.e. the “universal” reference patient) or not;
- whether VOD was more progressed in the originally-chosen reference (or 12th) patient of Cohort III, compared to the 1st patient in Cohort II or not.

In each case, the “universal” reference patient, i.e. the 1st patient in Cohort II, was diagnosed by the Haematologists to clearly have VOD progressed more severely; in fact, both the originally-chosen reference patients were diagnosed at the post-transplant stage to be VOD-free. The inter-graph distance between the 5th patient of Cohort I and 1st patient of Cohort II is computed to be 1.17; the distance between the 12th patient in Cohort III and the 1st patient in Cohort II is 2.43. We recall that the “universal” reference patient, i.e. the 1st patient of Cohort II has a score of 1 assigned to them. Then given that this “universal” reference patient had more severe VOD than the other two patients, the score of the 5th patient in Cohort I is -0.17, while that of the 12th patient in Cohort III is -1.43.

Indeed, in this form of learning, we do not retain the capacity for learing uncertainties on the VOD-score of the reference patients in Cohort I and III. This is why in Table 1, the entries under the column for the 95% Highest Probability Density credible regions is stated as “N.A.” for these two patients. Of course, for the 1st patient of Cohort II, who is the “universal” reference patient, the score is assigned as 1, leaving no scope for uncertainties on this score. This is again reflected in the table.

**Table 1:** VOD-scores learnt in the retrospective cohort, independently of the VOD status observed for a patient, indicated with a “Y” for onset of VOD, and “N” for no VOD onset, as identified post-transplant by the physicians. Score of the “universal” reference patient is set as 1 with no uncertainties, and all other scores are learnt relative to this score.

Mean of learnt score	Learnt 95% HPD	VOD status
0.11	[0.089, 0.31]	N
0.44	[0.29, .67]	Y
-0.069	[-0.24, 0.11]	N
-0.96	[-1.54, -0.37]	N
-0.17	N.A.	N
1	N.A.	Y
1.37	[1.34, 1.40]	Y
0.48	[0.43, 0.52]	Y
1.51	[1.46, 1.55]	Y
0.44	[0.38, 0.48]	Y
0.54	[0.49, 0.57]	Y
0.50	[0.46, 0.54]	Y
1.55	[1.51, 1.60]	Y
-1.18	[-1.19, -1.17]	N
-1.26	[-1.26, -1.24]	N
-1.74	[-1.75, -1.73]	N
-2.32	[-2.33, -2.306]	N
-1.55	[-1.55, -1.539]	N
-1.20	[-1.20, -1.186]	N
-1.45	[-1.46, -1.44]	N
-2.13	[-2.14, -2.127]	N
-1.58	[-1.587, -1.572]	N
-1.30	[-1.31, -1.299]	N
-1.18	[-1.19, -1.177]	N
-1.43	N.A.	N

So now that we have shifted the score for the 5th patient, (i.e. the originally-chosen reference patient in Cohort I), by -1.17, all other patients in Cohort I—whose originally learnt scores were learnt using a value of 1 for this 5th patient of this cohort—have their scores shifted by -1.17. The resulting scores, along with the uncertainties, are reported in Table 1.

Similarly, all patients in Cohort III have their scores shifted by -2.43, since the originally-assigned score of 1 on the erstwhile reference patient of this cohort (patient number 12), had their score shifted from 1 to -1.43. These updated scores of all in Cohort III are again reported in Table 1.

This way, we express the scores of all patients in the retrospective cohort, relative to the score of 1 that has been assigned to the arbitrarily-chosen “universal” reference patient for the whole cohort. Hereon, we speak only of the full retrospective cohort.

### 5.2 Learnt VOD-scores and interpretation

While we learn the VOD-score, interpreting that score to predict whether a patient will develop VOD after undergoing the transplant—or not—will be driven by calibration of our learnt scores against diagnosis of VOD status, which however is only possible post-tansplant, since VOD—if it develops in a patient—develops due to the transplant. (Here we recall that our aim is to predict, at the pre-transplant stage, the VOD-score of a prospective patient). We use the available information on the post-transplant VOD status of patients in the retrospective cohort, to cross-reference against our learnt VOD-scores for each such patient. Such cross-referencing informs on how to interpret our learnt VOD-scores, from the context of VOD status, i.e. whether the learnt score implies that VOD will onset in the patient, or not. Of course, our VOD-score informs on more than the binary VOD status; our predicted continuous VOD-score also informs on how intensely VOD will progress in a patient, after they have undergone the transplant.

In Table 1, we present the VOD-scores that we have learnt for each patient in the retrospective cohort, and we compare this to the presented VOD status of the corresponding patient, as observed by the physicians, after the transplant. Cross-referencing against the post-transplant VOD status, informs on the result that, a learnt mean VOD-score *S* ≤ 0.11 implies avoidance of onset of VOD. Any patient for whom the learnt mean VOD-score is >0.11 is noted to have developed VOD. Scoring with respect to the arbitrarily-chosen “universal” reference patient, allows us to maintain this cutoff mean score of 0.11 as the benchmark value, against which VOD onset is checked. If we take the uncertainties on the learnt scores into account, then we note that scores can maximally be 0.31 for the patient to not have VOD.

**Fig 3:**
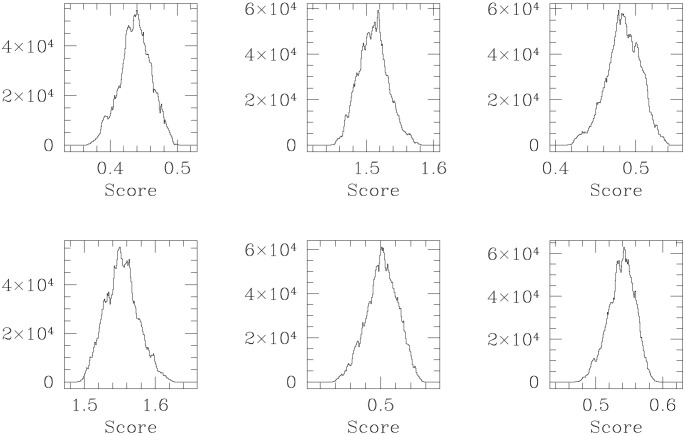
Histogram representations of the marginal posterior probability of VOD-scores of 6 retrospective patients, learnt given the data D_*δ*_. The marginal density is used to compute the 95% HPD on this patient’s VOD score, (tabulated in Table 1).

If the physicians were mistaken in the identification of VOD status in patients at the post-transplant stage, then we will be incorrect in our interpretation of 0.11 as the cut-off mean score, below which VOD status is negative—but if a prospective patient is learnt to attain a mean score above 0.11, they are interpreted to have developed VOD. Importantly, we note that the clinical identification of the severity of VOD development in the retrospective patients, concurred with the magnitude of our learnt VOD-score of the patient. That only (mean) scores learnt to be in excess of 0.11 are noted to correspond to patients identified by the Oncologists to have developed VOD, lends confidence in our learning, and in our formulation of the VOD-score variable, as a one-to-one parametrisation of VOD progress. In Fig 3 we display results of the MCMC-based learning of the scores of 6 retrospective patients using the inference discussed in Section 3.4.

### 5.3 VOD-score of a prospective patient

**Fig 4:**
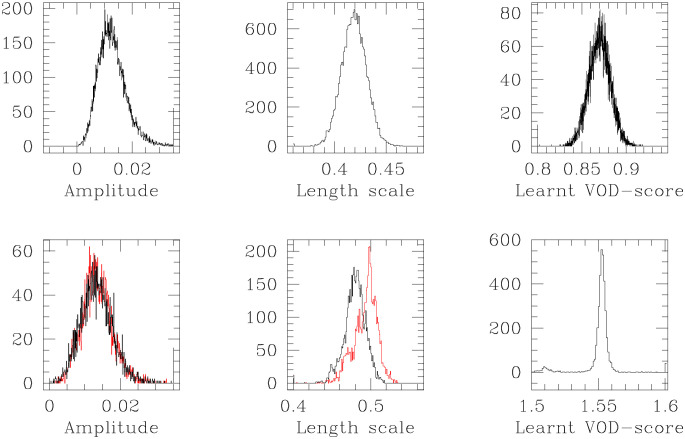
Histogram representations of learnt marginal posterior probability densities of VOD-score (right panels); length scale hyperparameter *ℓ* of the covariance kernel (middle panels); and amplitude parameter *a* of the kernel (left panels), given the training data, augmented by the observed pre-transplant vector of patient P-1 (lower panels) and patient P-4 (upper panel). The *ℓ* and *a* values learnt using the training set alone, are overplotted in red.

We learnt the VOD-score in 7 prospective patients; for each prospective patient, this learning was undertaken along with the learning of the length scale and the amplitude hyperparameters of the covariance kernel, (see Section 3). For these 7 prospective patients, (P-1, P-2, P-3, P-4, P-5, P-6, P-7), results of learning the VOD-scores within the learnt 95% HPD are shown in Table 2. In Fig 4, we plot the results of learning the VOD-score, the length scale hyperparameter *ℓ*, and the amplitude hyperparameter *a*, given the observed pre-transplant vector for prospective patient P-2 (in the lower panel) and prospective patient P-5 (upper panel).

**Table 2:** Relative VOD-scores learnt for 7 prospective patients. The predicted VOD status is tabulated in the 4-th column, while the VOD-status, observed as it develops in the patient post-transplant, is in the 5-th column.

Test patient ID	Mean score	Learnt 95% HPD	Predicted VOD status	Observed VOD status
P-1	1.551	[1.543, 1.562]	Y	Y
P-2	0.484	[0.475, 0.496]	Y	Y
P-3	0.996	[0.950, 1.045]	Y	Y
P-4	0.967	[0.949,1.033]	Y	Y
P-5	0.903	[0.849,0.901]	Y	Y
P-6	0.111	[0.100.120]	N	N
P-7	0.111	[0.102, 0.124]	N	N

#### 5.3.1 Advantages of risk prediction before transplant

Treatment with VOD prophylaxis of Defibrotide is expensive—at ∼$500 per vial, where 2–3 vials need to be administered daily, to attain proper prophylaxis. This is not affordable for all patients. The Oncologists in our team inform all their patients about the predicted risk of them developing VOD—with this risk quantified using our learnt VOD-score. All patients are informed about the relevance of the option of Defibrotide usage in their case, in the context of the predicted risk. For all patients there are other less expensive interventions possible, the evidence of efficaciousness of which are however anecdotal, distinguished from that of Defibrotide administration [15, 37]. Such less expensive, preventative interventions include fresh frozen plasma; Glutathione; Ursodexoycholic acid; N-Acetyl Cystiene infusion; Heparin infusion. So while all patients were offered these, Defibrotide administration is possible only for those who can afford the same. In other words, costs of Defibrotide typically prohibit universal administration of VOD prophylaxis at the pre-transplant stage, permitting usage of such pharmacological intervention only in the high-risk patients. Our work enables knowledge at the pre-translant stage, of a propsective patient’s risk of developing VOD post-transplant.

Of the five at-risk patients—as identified in our work—VOD prophylaxis administration was possible in P-2 at the pre-transplant stage; post-transplant, P-2 developed only mild VOD symptoms. The other four patients who were flagged with high VOD-scores at the pre-transplant stage, could not be covered by Defobrotide administration at the pre-transplant stage owing to affordability issues relevant to their circumstances. Amongst them, patient P-1 suffered from early demise, s.t. VOD status in them was inconclusive. Indeed, we had learnt a high risk of VOD onset in this patient, when they were at the pre-trasplant stage; learnt mean VOD-score was about 1.55 for P-1 at the pre-transplant stage. Again, P-5 went on develop mild VOD symptoms, where we had learnt a mean score of about 0.9 for them. P-3 did not develop VOD post-transplant, (we had learnt a score of about 1 for them), while P-4, (for whom we had learnt a score of 1 at the pre-transplant stage), went on to develop VOD, post-transplant. P-6 and P-7 did not develop VOD; our learnt mean scores for them ∼ 0.11.

#### 5.3.2 Prediction at input that lies outside convex hull of the training set

The learning of the score of test patient P-1 was tricky since pre-transplant thromboembolism was present for this patient, such a condition was not one that any of the retrospective patients had suffered from. In other words, our available training dataset could not enable any information to be gleaned on the relevance of thromboembolism to the VOD score that we aim to learn for any prospective patient, while the medical opinion of the Haematologist-Oncologists in our team was that this condition renders patients susceptible to VOD [38]. Since the training data that we employ, did not bear information on the effect of this condition on VOD-score, learning of the VOD-score of this patient was expected to be misguided, given the training set that we had access to. The learning of the VOD-score in this patient is then equivalent to demanding that we predict at an input (pre-transplant parameters) that lies outside the convex hull of the training set. Such prediction is equivalent to extrapolation, which—if robust—is a highly desirable property of any learning strategy.

To undertake the desired extrapolation within our Bayesian methodology, we enhance the information content affecting the learning of P-1’s score, by allowing for the prior information that thromboembolism renders P-1 more susceptible to VOD, than otherwise. The Haematologist-Oncologists in our team were moderately convinced about this prior. This led us to a Normal prior on the VOD-score variable *S*^(*test*)^ for patient P-1, where mean of this Normal was varied in the interval [0.5,1.15]—given that VOD-scores in this range correspond to moderate-to-high VOD severity in retrospective patients—and variance of this Normal prior is set s.t. this variance reflects the moderate levels of conviction of the medical practitioners amongst us. We used a variance of 0.3^2^, and thereafter relaxed the variance up to 0.5^2^ to check for prior-sensitivity of our learning. Priors defined by mean and variance values suggested above, consistently led to P-1’s VOD-score to converge to 1.55. We used widely different seed values of the sought score in our learning, to attain this result consistently.

## 6 Ranking pre-transplant variables by potency

In this section we discuss the potency of each of the (30) pre-transplant variables that populate the pre-transplant variable vector ***Y***; it is the relationship between ***Y*** and the VOD-score variable that we learn. Once the score *s*_*j*_ is learnt for the *j*-th patient in the retrospective cohort, we populate the training data $D={\left\{\left(s_{j},y_{j}\right)\right\}}_{j=1}^{N_{p}}$, where the vector ***y***_*j*_ of pre-transplant variables of the *j*-th patient are recorded, ∀*j* = 1, …, *N*_*p*_. Thereafter, using this data **D**, we non-parametrically learn the function ***f***(⋅), as a sample function of a Gaussian Process, (where ***Y*** = ***f***(*S*)). The question that we now ask, is about the ranking of the components of the pre-transplant vector variable ***Y***, by the influence that these variables *Y*_1_, …, *Y*_30_ have, on the VOD-score *S*.

To address this question we seek the *pdf* of the data variable $D_{Y}=\left\{Y_{1},\vdots ,\ldots ,\vdots Y_{N_{p}}\right\}$ built with the *d*=30-dimensional pre-transplant vector variables of the *N*_*p*_ patients in the retrospective cohort, corresponding to the respective design input, given the model parameters, and compare this to the density of another data variable $D_{Y-Y_{n}}$ and the design inputs, given the model parameters. Here $D_{Y-Y_{n}}$ is the data variable given as $\left\{Y_{-n,1},\vdots ,\ldots ,\vdots ,Y_{-n,N_{p}}\right\}$, ie. ***Y***_−*n*,*j*_ is the pre-transplant vector comprising all but the *n*-th component of ***Y*** in the *j*-th patient. Then $Y_{-n,j}\in R^{d-1}$, s.t. value of ***Y***_−*n*,*j*_ recorded for the *j*-th retrospective patient is ***y***_−*n*,*j*_, *j* = 1, …, *N*_*p*_; *n* = 1, …, *d* = 30. The density of the data variable, given the model parameters—which in our learning are the hyperparameters *ℓ* and *a* of the covariance kernel—is the likelihood. In fact we compute

$$\begin{array}{ccc} \gamma_{n} & ≔ & log(f_{D_{Y-Y_{n}},S_{1},\ldots ,S_{N_{p}}|\ell ,a}\left(D_{y-y_{n}},s_{1},\ldots ,s_{N_{p}}|\ell ,a\right))\\ & & -log(f_{D_{Y},S_{1},\ldots ,S_{N_{p}}|\ell ,a}\left(D_{y},s_{1},\ldots ,s_{N_{p}}|\ell ,a\right)), \end{array}$$ (1)

for *n* = 1, …, *d*. Here, the realisation of the data variable ***D***_***Y***_ is the the data **D**_***y***_ that comprises measurements of the pre-transplant variable vectors of *N*_*p*_ patients. Similarly, the data comprising measurements of all-but-the-*n*th-component of the pre-transplant variables is **D**_***y***−*n*_.

In the given data **D**, if the value of *γ*_*n*_ is more negative than that of *γ*_*n*^/^_, the model of the relationship between the observable VOD-score variable, and the observable pre-transplant variables *Y*_1_, …, *Y*_*n*−1_, *Y*_*n*+1_, …, *Y*_*d*_, is a worse than the model of the relation between *S* and $Y_{1},\ldots ,Y_{n^{/}-1},Y_{n^{/}+1},\ldots ,Y_{d}$, given the training data. In other words, the more negative is *γ*_*n*_, the effect of *Y*_*n*_ is more sorely missed in the model, given data **D**. However, if on removing *Y*_*n*_ from the model, likelihood improves, i.e. if *γ*_*n*_ > 0, it implies that the model is better without *Y*_*n*_ than with it included in the model. Thus, by varying across all *n*, we identify the pre-transplant variables by the order of their influence on the VOD-score, i.e. in the model of the relation between the score and the pre-transplant variables. Ranking of *Y*_1_, …, *Y*_*d*_, by such influence is tabulated in Table 3.

**Table 3:** Pre-transplant variables, ranked in order of influence on VOD-score, as identified by the difference in the *pdf* of the data comprising observations of the pre-transplant variable vector *Y* and that of the data on the vector *Y*_−*n*_ that comprises all-but-*n*th-pretransplant-variable, ∀*n* = 1, …, *d*, where *d* = 30 in our work. This difference in the density of the data variable built with ***Y***_−*n*_, and that built with ***Y*** of the *N*_*p*_ patients, given the model parameters, is referred to as “Difference in likelihood”, and tabulated in Column 2, in ranked order, for the *n*-th component of ***Y***, where the name of such component is mentioned in Column 1, under “Attribute”. The 3rd and 4th columns tabulate the difference in the learning of the model parameters *ℓ* and amplitude respectively, given data on ***Y***_−*n*_ and given data on ***Y***, where the corresponding omitted *n*-th component of ***Y*** is named in the same row of the 1st column. The difference caused in the posterior probability density of *ℓ* and amplitude, given the data on ***Y*** and that given data on ***Y***_−*n*_, is tabulated in the 5-th column, while the sum of the differences tabulated in the 2nd to the 5th columns, is noted in the 6-th column.

Attribute	Difference in likelihood	Difference in *ℓ*	Difference in amplitude	Difference in posterior	Sum of differences
ALL	-2.561	6.67×10^−5^	-0.002383	596.7	594.2
H/O Liver dis	-2.519	6.797×10^−5^	-0.002347	589.7	587.2
Dis-relapsed	-2.519	6.733×10^−05^	-0.00234	588.5	585.9
Diabetes	-2.518	7.104×10^−5^	-0.002345	589.5	587
GenMis-M:F	-2.513	6.886×10^−5^	-0.002331	586.5	584
GenMis-F:F	-2.513	7.036×10^−5^	-0.002338	588	585.5
Azathropine	-2.512	6.896×10^−5^	-0.00235	590.4	587.9
HLA match	-2.511	6.867×10^−5^	-0.002326	585.8	583.3
CMV-positive	-2.508	6.81×10^−05^	-0.00233	586.4	583.9
Gen-Mis-M:M	-2.507	6.581×10^−5^	-0.002324	585.2	582.7
6-Mercatoprine	-2.506	6.753×10^−5^	-0.002333	587.1	584.6
Myelotarg	-2.506	6.702×10^−5^	-0.002332	586.8	584.3
Aplastic Ane	-2.506	6.999×10^−5^	-0.00234	588.3	585.8
Cytarabine	-2.505	6.956×10^−5^	-0.002335	587.4	584.9
GenMis-F:M	-2.504	6.641×10^−5^	-0.002327	585.9	583.4
CML	-2.503	6.937×10^−5^	-0.002326	585.7	583.2
PulmonaryDysf	-2.502	6.612×10^−5^	-0.002329	586.2	583.7
AML	-2.5	6.748×10^−5^	-0.002327	585.8	583.3
CMV-reactive	-2.5	6.762×10^−5^	-0.002311	582.7	580.2
CardiacDis	-2.497	6.796×10^−5^	-0.002327	585.9	583.4
ABOMismatch	-2.495	7.26×10^−5^	-0.002314	583.3	580.8
HepaticDysfun	-2.488	6.996×10^−5^	-0.0023	580.6	578.1
Dis-remission	-2.478	6.749×10^−5^	-0.002299	580.5	578
Other^1^	-2.463	5.996×10^−5^	-0.002278	576.3	573.9
Myleofibrosis	-2.463	5.996×10^−5^	-0.002278	576.3	573.9
Hypertension	-2.458	6.868×10^−5^	-0.002283	577.2	574.8
Gender	-2.049	3.499×10^−5^	-0.00184	488.6	486.5
Age	-2.049	3.499×10^−5^	-0.00184	488.6	486.5
FerritinPre-trans	-1.457	0.0001499	-0.001198	353.5	352.1
CRPPre-trans	2.89	-2.474×10^−5^	0.004648	1245	-1243

^1^ Diseases other than Acute Myleoid Lukemia (AML), Acute Lymphoblastic Leukaemia (ALL), Chronic Myeloid Leukaemia (CML), Aplastic Anemia and Myleofibrosis are referred to as “Other” in Table 3.

### 6.1 Discussion

In this section, we have discussed a method to rank the considered pre-tramsplant variables by the strength of their effect on VOD progression. This is an important exercise to undertake; [18] say, “Recognition of VOD/SOS risk factors helps expedite treatment”; [6] report on VOD risk factors, as part of a review on VOD incidence and diagnosis. We identify H/O liver disease—and hepatic dysfunction as a co-morbidity—to potently influence VOD progression. Indeed, [2, 4, 11, 12, 39] also report pre-existing liver disease as a risk factor of VOD. Existing pulmonary dysfuction is also concluded to be a risk factor in our work, as was suggested by [40].

Again positive CMV serology is noted to be a risk factor by [41], and we find this to influence VOD onset. In our work, advanced age is noted to be a relevant risk factor, as is suggested by [4, 42]. We find high values of pre-transplant ferritin to be less influential a factor for VOD onset than the pre-transplant attributes discussed above, while [43] forward this to be a relevant risk factor. In terms of transplant parameters, we identify HLA mismatch to be an important factor of influence, in agreement with [11]. Additionally, gender mismatch is found to be more potent than ABO mismatch in our work, in influencing VOD progression. Then again, in terms of pre-transplant medication, Azathioprine; 6-Mercaptopurine; Myelotarg; and Cytarabine are found to bear influence on VOD progression—in that order, given our sample; indeed, [44] have suggested pre-treatment with Myelotarg to affect VOD, while [45, 46] report VOD and sinusoidal dilation as linked to treatment with Azathioprine.

Our work finds the underlying cancers to be relevant, namely, ALL, Aplastic Anemia, CML, AML, Myleofibrosis, and “Other” (defined within the table above)—in order of influence; [47, 48] have reported on the relevance of Advanced malignancy, Acute leukemia and Neuroblastoma. We find cancer status to be a relevant risk factor, with relapsed state of the disease—over remission—noted to affect VOD progression, relatively more strongly. The parameters of the transplant procedure are not pre-transplant parameters, and are therefore not included in our ranking of risk factors. Our results—including the addressing of co-morbidities as influencing VOD—are likely to be sample-bounded. The method of detection of the relative potency of risk factors is the intended deliverable of our work; results of illustration of this method on our sample is reported in Table 3.

## 7 Conclusions

We present reliable learning of scores that inform on how virulently a disease will afflict a patient at the pre-onset stage, given their pre-onset parameters. In the context of the disease VOD, we learn the VOD-score of each in a cohort retrospective patients. We learn this score, using the multivariate time series data on physiological parameters of each patient; data of such patient physiological parameters is employed to generate the graphical model of the patient’s evolving physiology. Under the assumption that VOD severity in one patient compared to that in another, is dependent on the difference between the correlation structures of the temporal evolution of the physiological parameters of this pair of patients, we learn the score of each patient in the cohort, relative to an arbitrarily-chosen reference patient. This assumption stems from the understanding that these relevant physiological parameters that oncologists record for a patient from 8 days before, to 18 days after the bone marrow transplant, are s.t. the inter-physiological-variable correlation structure, as manifest in the graphical model of the post-transplant time series data of such physiological parameters, is contributed to by VOD progression. Such contribution is corroborated by the medical opinion of Haematologist-Oncologists.

However, the linear dependence between the difference in a pair of VOD-scores, and the (Hellinger) distance between probability distributions of the corresponding graph variables, is a model we use, for the definition of the VOD-score. It is our learning of the graph as a random variable that allows learning of its posterior probability, allowing for the Hellinger distance to be computed, yielding the score in turn. Also, we learn the vector-valued functional relation between the VOD-score and the pre-transplant vector variable, implying that the input VOD-score is learnt—instead of predicted—for a new patient, using their pre-transplant variables.

Our learnt pre-transplant VOD-scores of prospective patients, enabled the physicians to consider administration of VOD prophylaxis to at-risk patients, at the pre-transplant stage. For the five out of the seven prospective patients whose VOD-scores learnt at the pre-transplant stage, exceeded 0.11, the Haematologist-Oncologists in the team designed their respective pre-transplant treatment regimen to preferably include VOD prophylaxis with Defibrotide. On the other hand, the treatment regimen of patients with pre-transplant VOD-scores that indicated lack of susceptibility to VOD, precluded Defibrotide.

From a method development point of view, our method has the advantage that we can learn the VOD score of a prospective patient whose inputs lie outside the convex hull of the training set. Additionally, our methodology is a generic one that can be implemented to learn the progression score of other diseases at the pre-onset stage. Curently the method is being employed to learn the score of movement recovery in patients who are undergoing physical rehabilitation, following a movement-impeding critical illness; recovery of one patient relative to a reference patient is parametrised in this application, as the distance between the graphs realised from the undertaking of relevant exercises by the two patients. This analysis is paving the way for the learning of recovery trajectories in patients, as well as for the identification of the optimal treatment regimens for selected patient groups. Once such movement recovery scores are learnt, the relation between the pre-illness parameters and score variable will be learnt, to thereby predict the recovery trajectory for a patient with given pre-injury parameters.

## Supporting information

S1 Data (XLSX)
